# Editorial

**Published:** 1851-05

**Authors:** 


### THE MEDICAL EXAMINER.

PHILADELPHIA, MAY, 1851.

### RANK OF MEDICAL OFFICERS IN THE U. S. ARMY.

We have before us the "Report of a board of army officers appointed by the President upon so much of the resolution of the House of Representatives of July 18, 1850, as relates to rank and command within the army," dated Washington, November 27, 1850. The board consisted of Major General Winfield Scott; Brevet Major Generals Th. S. Jessup and John E. Wool; Colonel J. B. Crane; Lieut. Col. E. A. Hitchcock; Deputy Paymaster General Benj. F. Larned; and Surgeon T. G. Mower.

The report of the board is in form of a bill, or project of a law. The first section sets down what shall be the "titles and grades of army rank, or rank in the line of the army," which are in the following order: viz. 1, Major General; 2, Brigadier General; 3, Colonel; 4, Lieutenant Colonel; 5, Major; 6, Captain; 7, First Lieutenant; 8, Second Lieutenant; 9, Cadet; 10, Ordnance Sergeant; 11, Sergeant Major; 12, Quartermaster Sergeant; 13, Principal Musician; 14, Orderly, or First Sergeant; 15, Sergeant; and 16, Corporal. The last is entitled to command all privates, musicians, artificers and the like. This section provides that all persons attached to the army who are not commissioned, shall be subordinate to any commissioned officer of any of the staff corps, including of course, officers of the medical and pay department, though "without army rank."

According to this scheme, the line of the army consists of sixteen official grades, from Corporal to Major General. But the line of the army could not sustain itself alone; it requires the aid of a class of military men, called the staff, which consists of several distinct departments and corps. The commissioned officers of the line of the army, (attached to regiments of dragoons, mounted riflemen, artillery and infantry,) at the close of the year 1849, numbered 585; and at the same time the staff of the army included 324 commissioned officers. These figures show the relative proportion which the line and staff bear to each other. The general staff of the army consists of the Adjutant General's department; the inspector's department; judge advocate of the army; quarter master general's department; commissary general's department; medical department; pay department; besides the corps of engineers, and that of the topographical engineers; the ordnance department and military store-keepers. The special duties of these several departments are all essential to the efficiency of the line: they embrace rolls, registers of service, feeding, clothing, lodging, paying; curing when sick or hurt; surveying routes and camp grounds; building forts and barracks, and taking care of depots of military stores of various kinds, &c. &c.

These staff departments are constituted of several grades; and each is organized in itself after the manner of the line of the army: for example, the corps of engineers consists of six grades of commissioned officers, namely, colonel; lieutenant colonel; major; captain; first lieutenant; second lieutenant. These titles and grades in the engineer staff, (composed in fact of architects whose business is to build for military or warlike objects,) bear a similar relation to each other, subordinately, as the same titles and grades in the line of the army. In a word, the rank of an engineer amongst engineers is precisely the same thing, as the rank of a line officer amongst officers of the line, in its meaning and application. But as the term rank has come to mean in military communities, relative position as well as the degree or limits of authority an officer may exercise; and as it would lead to confusion, were it admitted that grade and title impart power which may be exercised, at the will of the officer, for example, either as a major of infantry, or of artillery, or of dragoons, or of engineers, according to circumstances, different qualities of rank have been established. The position of an officer in the line of the army is termed his *lineal* rank; the position of an officer in the engineer, or other corps is termed his *staff*-rank. It is established that a major of engineers (military architects), cannot in virtue of his title of major, exercise authority as a major in the artillery, or infantry, or in any department of the line, or staff; but it is also understood that no major whose commission is junior in the line, can control the major of engineers, except under the special instructions of a common superior. Yet in point of military respectability the title of "major" is the same, whether used in the line or staff; a major of engineers is on a level with a major of artillery, or a major of any other arm or branch of the army. The authority which enures to title and grade is restricted to the department or corps to which they belong. But all men in an army are military men, no matter what may be their vocation; a military tone, a military spirit, a military sensibility or sense are equally diffused and common to all, in the same manner as a Christian sensibility or perceptivity pervades and is common among all civil communities or societies in the United States. No matter what may be his trade or vocation, every citizen of our country is assumed to be a Christian; and so, every member of an army, no matter what may be his special employment, is presumed to be a *militaire.*

But this report strives to except from the rule and to deny that physicians, who are members of the army, can have military tone or perceptivity. The line officers would have physicians in the army placed in a *caste* position relatively to the army, such as Jews, or infidels, commonly hold in strict Roman Catholic societies or countries. The science of medicine seems, in the opinion of the line, to degrade its votaries, much in the same manner as the Jewish creed degrades its votaries in the estimation of many Christians. It is against this purely *caste* distinction that medical officers contend; they insist that a major in the medical department is entitled to as much respect and consideration in the army and out of it, as a major in the engineer department, subsistence department, clothing department or any other, and that they are not disqualified, in consequence of exercising the profession of medicine and surgery, from participating in the general and common military spirit which pervades the army. They contend that the profession of medicine does not disqualify them from serving on boards, councils, or courts-martial; but they are willing nevertheless to be exempt from such duties, in the same manner that magistrates, judges, physicians, counsellors at law, and officers of the army and navy, and some others in civil life, are exempt from serving on juries. They contend that surgeons in the army are military men, and that their commands are therefore military; because *military command* consists essentially in a legal right to exact obedience, under the penalties provided by military laws, without regard to the nature of the act ordered to be done. On this principle they claim rank, which is defined to be the relative position of the members of a military community to each other. We have already explained the meaning of the terms lineal rank and staff rank. There is still another quality of rank, termed *assimilated* rank, which is defined to be the relative position of members of the staff corps to officers of the line, and to officers of different staff corps.

The report of the board before us, in the first section, defines the lineal rank of the army, and, in the second section, admits staff and assimilated rank for all except the medical and pay departments. The seventh section proposes to fill vacancies, or in a word to make promotions in certain staff corps by selections from the whole army; but the proposition has been significantly rebuked by a clause in the law recently enacted, making appropriations for the support of the army, which provides "That all promotions in the staff departments or corps shall be made as in the other corps of the army."

The fifth section proposes that officers of the medical and pay departments shall be subordinate, shall always be virtually of inferior rank, to any line officer accidentally in command of a post or detachment, no matter what may be the grade of such line officer. No such exception is proposed against the rank of officers of any other staff department of the army. The assimilated rank of commissaries, quarter masters, engineers, &c., is never made contingent in its operation on circumstance; a major of the quartermaster's or engineer's department remains a major, although the command of a post or detachment may have devolved upon a captain or first lieutenant of the line. It is not perceived why an invidious distinction should be made against medical officers in this respect.

The ninth and last section proposes to repeal the laws which confer rank on medical officers, but not to deprive those now in service of their present rank; all officers hereafter appointed in the medical department of the army are to be commissioned without any rank or position whatever. If the medical officers now in the army can retain the rank which the law gives them without injury or inconvenience to the service, it is absurd to suppose that those hereafter appointed cannot enjoy the same rank, without risk of detriment to the discipline of the army.

For the various propositions of this report, no reasons whatever are assigned; but we have the protests of the staff-officers against them. That of Surgeon Mower speaks for itself, and is commended to the special attention of the medical profession generally, the members of which should give their influence in obtaining and securing for their brethren in the army and navy a just position. It is to be hoped that no physician in the United States will cast his vote in favor of any candidate for the presidency or other political office, who is known to be hostile to or prejudiced against the just claims of medical men to be protected by law, like other citizens. The mental capacity and impartiality of a man may be doubted, if he is found to regard physicians as men of inferior caste; or less respectable on account of their vocation.

If the opposition to assigning medical officers rank in the army and navy were based upon any sound reason, there would be no difficulty in stating such reason or argument. But thus far not a single argument of any weight whatever has been brought forward by gentlemen of the line, either of the army or navy. The whole of the opposition rests on prejudice against the medical profession, more than against its members who generally enjoy a high degree of social popularity in both services, in spite of the vocation, which would be more justly appreciated by the masses if it in any manner represented property, or value. If medical prescription saved the patient from the expenditure of dollars, instead of relieving him from pain or rescuing him from death, its worth would be more palpable, and it would be cheerfully paid for at full price.

WASHINGTON, D. C., *November* 27, 1850.

The undersigned, dissenting from the views of a majority of his colleagues, as indicated in several sections proposed for enactment, feels constrained to enter this his protest against them, and respectfully to request that it may accompany the report of the majority of the army board.

He dissents from the proposition to allow staff officers to assume command of troops in any case where there is an officer of the line of the army present for duty, unless such staff officer be especially assigned to such command by the President of the United States, or by a general officer commanding in the field—this, on the ground that the staff officer should not be diverted from his appropriate duties, that his pay and emoluments are superior to those of the line officer, and that this inequality would be still further increased by the allowances incidental to his entering on a command.

He dissents also from the last clause of section 5, which is in the following words: "And when they (officers of the medical and pay departments) chance to be at a post or with a detachment commanded by a junior officer, they shall not absent themselves from the post or detachment, except in urgent cases, and then not without notifying the commanding officer, though of inferiorrank, of their intention so to do;" because it would unnecessarily establish an invidious distinction between certain staff departments and corps, and other co-ordinate staff departments, to wit: the medical and pay, and would, as a necessary consequence, create an odious *caste* among the officers composing the staff of the army. Under existing law and regulations, an officer of the staff, to whatever corps he may belong, cannot assume the command of a body of troops over an officer of the line, "except by special assignment;" nor can any officer of inferior rank in the line command his senior in the staff. In this last respect the officers of all the staff departments and corps enjoy the same immunity, and are upon an equal footing. The clause referred to not only breaks down this just equality between the officers of the staff of the army, but singles out those of the medical and pay departments as the special exception to the rule. Moreover, while it is proposed to remove the legal and executive disabilities to which other staff departments and corps are subjected, and even to extend their privileges, the strictly *protective* rank of officers of the medical and pay departments has been so hampered that they are prohibited from a walk out of camp or garrison "without notifying the commanding officer, though of inferior rank, of their intention so to do."

If any conflicting case has occurred which to the minds of the ma jority of the board renders it necessary that the laws conferring *protective* rank upon the officers of the medical and pay departments should be thus restricted, it should be borne in mind that the responsibility of two unaccommodating tempers ought not to be thrown upon one, and that the difficulties resulting therefrom, more imaginary than real, should not be charged to him alone.

Whatever may be the principles on which they are based, the working of no human institution is perfect; nor is a military organization which aims at utopian results an exception. It may be worthy of consideration, therefore, whether a better incentive to the performance of their respective duties by the officers of the medical and pay departments (already subject to all the rules and articles of war, and to all the liabilities and penalties which they impose) could not be found in a high moral and professional principle, rather than in subjecting them to invidious and humiliating distinctions, by restricting their movements and their absence from post, even for half an hour, without previous notice to the commanding officer, though of inferior rank.

The proposition to make, contrary to existing laws, a senior medical officer virtually subordinate to a junior officer of the other branches of service who may chance to be in command at a post or station, is based on theoretical rather than on practical principles. Their duties being altogether distinct, there is actually no necessity for the one interfering with the other; and it may be remarked that the less this is done the better is it for the interest of the service. Experience may be confidently appealed to to prove that the medical officer is seldom interfered with by a commanding officer of well-regulated mind and of experience. And, in illustration, I may state that, previous to the present organization of the medical department, I served for a period of six years as surgeon to the 6th regiment of infantry, commanded by Colonel (Brigadier General) Atkinson, confessedly one of the best disciplinarians of the army, and, without any solicitation on my part, he voluntarily extended to me the privilege of absenting myself from post temporarily at my own discretion, on the practical and common-sense ground that I could best judge when my services with the command could be spared, and with the understanding that I was held strictly responsible for the performance of my appropriate duties. It gives me pleasure to add the testimony of one of the members of this board, an experienced and successful commander, that a similar practice obtained in his last command.

In determining the military privileges of the officers of the different staff departments and corps relatively with those of the line of the army, there is a manifest propriety in inquiring which of the former is more invariably associated on duty with the latter in garrison and camp, and most participates with them in the hardships and privations incident to the military service. Without hazard of contradiction, it is confidently asserted that it is the *medical department* of the army. On the field of battle, too, where blows are dealt, but cannot consistently with duty and professional engagements be returned, who are more exposed to the missiles of the enemy than the medical officer ?*

He protests also against section 9, which not only withholds all manner of rank from future medical appointments, dooming them to military outlawry, but likewise places an impenetrable barrier between them and their professional brethren now in the department. It is vouchsafed to the latter that they may retain the humble rank they now enjoy—diluted and qualified, to be sure ; but for the former there is no ray of hope afforded : they must enter the department, if their pride will allow them to seek the service, a degraded *caste,* not merely among the officers of the line and of the privileged staff corps, but also in their own branch of the service.

The last clause of section 8 of an act approved February 11th, 1847, provides that the rank of the officers of the medical department of the army shall be arranged upon the same basis which at present determines their pay and emoluments : " *Provided,* That the medical officers shall not, in virtue of such rank, be entitled to command in the line, or other staff departments, of the army." This clause, which it is proposed by section 9 to repeal with reference to all future appointments to the medical department, allows only a modified or protective rank to a class of officers confessedly as select and as meritorious as any other in the army. Previously to its enactment, it will be recollected what was the humiliating position assigned to the officers of the medical staff by paragraph 5, general regulations for 1841. Under this paragraph, a medical officer of twenty years' service might be (and frequently was) required to yield precedence on courts martial and boards to a junior officer, perhaps a brevet lieutenant. To obviate a contingency so humiliating to the officers of the medical department and to give them a fixed position which would not be subject to the *fluctuating changes of army regulations,* a legal provision was asked for and obtained. Under its benign operation and the fostering care of

* *Number of officers of the staff of the regular army killed, wounded, and died of wounds in the several conflicts during the war with Mexico, derived from the Adjutant General's report to the Secretary of War.*

| | Killed. | Wounded. | Died of Wounds. |
|---|---|---|---|
| Adjutant general's department, | 1 | 0 | 0 |
| Inspector general's department, | 0 | 0 | 0 |
| Quartermaster general's department, | 1 | 0 | 0 |
| Subsistence department, | 0 | 0 | 0 |
| Pay department, | 0 | 0 | 0 |
| Medical department, | 0 | 2 | 1 |
| Corps of Engineers, | 0 | 6 | 0 |
| Corps of topographical engineers, | 0 | 3 | 1 |
| Ordnance department, | 0 | 2 | 0 |

In addition to the foregoing, *two* officers of the "general staff" are reported to have been wounded in the battle of Buena Vista ; but it is not specified whether they were *general* or *staff officers,* and, if the latter, to what particular department or corps they belonged.

the government, aided by the salutary restrictions guarding appointments to the medical department, it has attained an eminence in personal and professional standing alike beneficial to the army and creditable to themselves. The young professional *élite* of the country have been attracted to the department, and will continue to be so as long as they shall be assured that their professional and personal merit shall be properly appreciated in the military service.

Then why this retrograde movement, and why jeopard the ability for usefulness to the army, which the medical department now possesses? In the name of humanity, the undersigned humbly trusts that no act may be done tending to lower the standard of professional excellence now happily existing in the medical ranks of the army.

T. G. MOWER, Surgeon United States Army.

---

### NASHVILLE JOURNAL OF MEDICINE AND SURGERY.

We have just received the first number of this new aspirant for professional favor, edited by Professor Bowling, and extend to it the right hand of fellowship with our best wishes for its success. We would call the attention of the editor to an article, in the January number of the American Journal of Medical Sciences, on the cod liver oil by *Dr. J. J. Levick* of this city; and whilst we do not desire in the least degree to underrate the valuable contribution of Dr. Yandell, which appears in this first number, we would claim for our townsman "the honor of being the first to convey to the medical public of this country, a detailed and scientific account of the GREAT REMEDY in tuberculous disease." In the edition of Dr. Gerhard's treatise on the diseases of the chest, published in 1850, will also be found the author's experience in its use in the Pennsylvania Hospital in tuberculous disease. "Suum cuique tributo."

